# Complete axillary dissection without drainage for the surgical treatment of breast cancer: a randomized clinical trial

**DOI:** 10.6061/clinics/2017(07)07

**Published:** 2017-07

**Authors:** Ruffo Freitas-Junior, Luís Fernando Jubé Ribeiro, Marise Amaral Rebouças Moreira, Geraldo Silva Queiroz, Maurício Duarte Esperidião, Marco Aurélio Costa Silva, Rubens José Pereira, Rossana Araújo Catão Zampronha, Rosemar Macedo Sousa Rahal, Leonardo Ribeiro Soares, Danielle Laperche dos Santos, Maria Virginia Thomazini, Cassiana Ferreira Silva de Faria, Régis Resende Paulinelli

**Affiliations:** I Gynecology and Breast Unit, Hospital Araújo Jorge, Goiás Anticancer Association, Goiânia, GO, BR; II Breast Program, Department of Gynecology and Obstetrics, School of Medicine, Federal University of Goiás, Goiânia, GO, BR; III Department of Pathology, School of Medicine, Federal University of Goiás, Goiânia, GO, BR

**Keywords:** Breast Cancer, Breast-conserving Surgery, Lymph Node Excision, Drainage, Postoperative Complications

## Abstract

**OBJECTIVE:**

This randomized clinical trial evaluated the possibility of not draining the axilla following axillary dissection.

**METHODS:**

The study included 240 breast cancer patients who underwent axillary dissection as part of conservative treatment. The patients were divided into two groups depending on whether or not they were subjected to axillary drainage. ClinicalTrials.gov: NCT01267552.

**RESULTS:**

The median volume of fluid aspirated was significantly lower in the axillary drainage group (0.00 ml; 0.00 – 270.00) compared to the no drain group (522.50 ml; 130.00 - 1148.75). The median number of aspirations performed during conservative breast cancer treatment was significantly lower in the drainage group (0.5; 0.0 - 4.0) compared to the no drain group (5.0; 3.0 - 7.0). The total volume of serous fluid produced (the volume of fluid obtained from drainage added to the volume of aspirated fluid) was similar in the two groups. Regarding complications, two cases (2.4%) of wound dehiscence occurred in the drainage group compared to 13 cases (13.5%) in the group in which drainage was not performed, with this difference being statistically significant. Rates of infection, necrosis and hematoma were similar in both groups.

**CONCLUSION:**

Safety rates were similar in both study groups; hence, axillary dissection can feasibly be performed without drainage. However, more needle aspirations could be required, and there could be more cases of wound dehiscence in patients who do not undergo auxiliary drainage.

## INTRODUCTION

The complications directly associated with the surgical treatment of breast cancer involving lymphadenectomy have been well established. They include seroma (the most common complication), hematoma, infection of the surgical wound, skin necrosis, winged scapula and lymphedema of the arm 1-4. In addition, some patients complain of considerable discomfort and difficulty with shoulder movement 5.

The systematic use of continuous suction drainage may reduce postoperative morbidity 3. Several other approaches have been tested in an attempt to decrease the accumulation of serous fluid or seroma formation, including reducing the duration of drainage 6, the use of sclerosants 7 or fibrin glue 8 at the surgical site, and the obliteration of dead space in axillary dissection 3,9,10.

Axillary drainage has become a subject of debate in recent years since it tends to prolong hospitalization times 11 and creates discomfort, exerting a negative psychological impact on patients who have undergone this treatment 5,12,13. Drainage is also associated with limitations to activities of daily living that may include difficulty in dressing and problems with sleeping 13. Consequently, some studies have been conducted to evaluate the possibility of not draining the surgical site following axillary dissection 9,11,12. However, controversy persists regarding the need to use continuous drainage 9, with the debate being mainly motivated by doubts that not performing drainage could increase the rate of complications, particularly in patients undergoing complete axillary dissection (levels I, II and III).

The primary objective of the present study was to investigate the safety of not draining the axilla. The evaluation was conducted by comparing the total number of breast cancer patients who did not develop complications following conservative surgery with complete axillary dissection according to whether or not axillary drainage was performed. The number of needle aspirations, the volume of fluid drained or aspirated and the total volume of fluid produced were compared between the two groups as a secondary objective.

## PATIENTS AND METHODS

The internal review board of the Federal University of Goiás teaching hospital approved the protocol of the present study under reference number 017/2000. The procedures were in accordance with the ethical standards of the responsible committee on human research and with the Helsinki Declaration of 1975, as revised in 1983. ClinicalTrials.gov: NCT01267552. Following approval, 240 patients with breast cancer were included in the study and submitted to conservative surgery with axillary dissection. Only patients who voluntarily agreed to participate in the study and signed the informed consent form were enrolled.

Sample size was calculated based on the number of complications detected in a pilot study conducted previously in this same institute. In that pilot study, the safety rate (i.e., absence of complications) for patients who were not submitted to axillary drainage was 72%. Taking into consideration a possible difference of 12% between the groups, with α=0.05 and β=0.20, a total of 202 patients were required for the study. The possibility of losses was estimated at 19%; therefore, the total number of patients to be enrolled to the study was increased to 240, a number intended to be distributed equally between the two groups.

### Inclusion and exclusion criteria

Female patients with clinical stage I or II breast cancer were included in the study. The recommendation for all these patients was conservative surgery, including a wide resection and complete axillary dissection (levels I, II and III). The procedures were to be performed either at the Araújo Jorge Hospital (ACCG) or at the university teaching hospital (UFG). Patients were only considered for inclusion if they fulfilled the inclusion criteria for both groups and if they agreed to participate in the study. Patients with diabetes were excluded.

### Randomization

Randomization was performed by means of a computer-generated list of consecutive random numbers. The information regarding the group to which each patient had been allocated was given to the surgeon minutes prior to the start of the surgical procedure.

### Breast conservation criteria

Conservative surgery was an option when the patients’ tumors were no greater than 4 cm in diameter. In the case of small breasts, the maximum diameter of the tumor that would permit conservative surgery to be performed was 2 cm. Although the simultaneous presence of multicentric foci or contralateral tumors did not *a priori* constitute a contraindication to conservative surgery, these were assessed on a case-by-case basis by the surgeon to determine whether this procedure was appropriate.

### Surgical technique

Conservative breast cancer treatment consisted of excising the tumor, taking care to leave wide margins, and then performing complete axillary dissection, including levels I, II and III.

After performing the cutaneous incision, the lymph nodes and all the adipose tissue located in the lower anterior portion in relation to the axillary vein were removed from its entry into the thoracic wall up to the anterior edge of the latissimus dorsi muscle. The pectoralis minor muscle was preserved at the surgeon’s discretion. The thoracodorsal nerve and the long thoracic nerve were preserved.

### Axillary drainage

In the group of women randomized to drainage, a continuous suction drain was installed in the surgical site before the wound was closed. The drain was exteriorized through a counter-opening and attached using non-absorbable suture thread. Inside the surgical wound, the drain was placed in such a way to drain the region overlying the pectoralis major muscle, the axillary hollow and the edge of the latissimus dorsi muscle. The external extremity of the drain was connected to a continuous suction system.

### Follow-up of the women in the drainage group

In this group of patients, the fluid was removed from the reservoir bulb twice daily, and its volume was recorded on a chart specifically designed for this purpose. In the ward, dressings were applied according to the standard procedures in the institute until the patient was discharged on the second postoperative day, at which time she was referred to the outpatient clinic for follow-up. Between the fifth and seventh postoperative days, the drain was removed. Whenever necessary, needle aspiration was performed, and the volume of fluid aspirated was recorded. The patients were then evaluated weekly, and any complications occurring up to the 30^th^ postoperative day were recorded.

### No drain group

In the group of women in whom drainage was not performed, needle aspiration was carried out whenever necessary up to the time of their discharge from hospital. The patients were advised to return once a week for assessment and for seroma aspiration as required. As in the drainage group, the volume of fluid aspirated was recorded, as were any other complications.

### Definition of the variables

Age: The patient’s age at the time of breast cancer diagnosis.

Tumor size: Measured across the largest diameter at the time of diagnosis.

Clinical stage: The clinical staging of the disease was defined in accordance with the International Union Against Cancer TNM classification.

Number of lymph nodes resected: The number of lymph nodes histologically identified in the surgical specimen obtained at axillary dissection.

Axillary lymph node status: Defined in accordance with the number of lymph nodes histologically affected by neoplastic cells.

Histological type: Defined in accordance with the World Health Organization (WHO) classification.

Seroma formation: Seroma was defined as any accumulation of serous fluid clinically detected beneath the flaps or in the axilla following surgery.

Volume of fluid drained: The total volume of fluid aspirated by the suction drain only for the patients in the drainage group, expressed in milliliters.

Volume of fluid aspirated: The total volume of serous fluid aspirated by needle aspiration, expressed in milliliters.

Number of needle aspirations: The number of times needle aspiration was required to drain the seroma.

Total volume drained: The sum of the volume of fluid drained and aspirated, expressed in milliliters.

Infection of the wound: The formation of cellulitis and/or accumulation of purulent secretion in the surgical area were considered indications of an infection in the surgical wound.

Wound dehiscence: Wound dehiscence was defined as the opening of the skin including exposure of the underlying tissue prior to wound healing.

Necrosis: Necrosis was defined as the presence of blackening areas on the skin flaps caused by deficient vascularization in the region.

Safety rate: The total number of patients with no complications divided by the total number of patients in each group.

### Statistical analysis

Means (± standard deviation) and a Student’s t-test were used for the variables with a normal distribution. In the case of numerical values with a non-normal distribution, the medians, interquartile ranges (IQR) and a Mann-Whitney test were used. For categorical variables, a chi-squared test (χ^2^) or Fisher’s exact test was used, as appropriate.

In the case of complications, the relative risks of each complication were analyzed together with their respective 95% confidence intervals. The significance level was set at 5%. The statistical software program SPSS (Chicago, IL, USA) was used throughout the statistical analysis.

## RESULTS

Of the 240 patients randomized, 119 were allocated to the axillary drainage group and 121 to the no drain group. The mean age of the patients was 49.64±11.93 years. The median tumor size was 30 mm (range 20 - 40 mm). The median number of lymph nodes resected was 17 (range 14 - 21). The number of histologically compromised lymph nodes ranged from 0 to 34, with a median of 0 (IQR: 0 - 2).

The most frequent histological type was invasive ductal carcinoma, which accounted for 215 cases: 104 in the drainage group and 111 in the no drain group. There were 8 cases of lobular carcinoma in women in the drainage group and 3 in the no drain group. Other histological types were found in the remaining 14 patients, with 7 in each group. No statistically significant differences were found between the two groups with respect to the distribution of histological types (χ^2^=3.08; *p*=0.68). Regarding the degree of tumor differentiation, grade II was the most common, accounting for 143 cases (59.6%), followed by grade III with 40 cases (16.7%), undetermined grade with 33 cases (13.7%) and grade I with 24 cases (10%). Analysis of the distribution of the histological grades also showed no statistically significant differences between the groups (χ^2^=1.03; *p*=0.79).

The two groups were similar with respect to the other control variables including age, tumor size, number of lymph nodes resected, and the number of compromised lymph nodes (Table 1).

The median volume of fluid drained was 0 ml (range 0 - 2,040 ml). The median volume of fluid aspirated was 240 ml (range 0 - 11,998 ml). The median number of aspirations was 3.00 (range 0 - 48). The median overall volume of all the fluid drained or aspirated was 649.00 ml (range 0 - 11,998 ml). Table 2 compares the two study groups.

**Figure 1:**
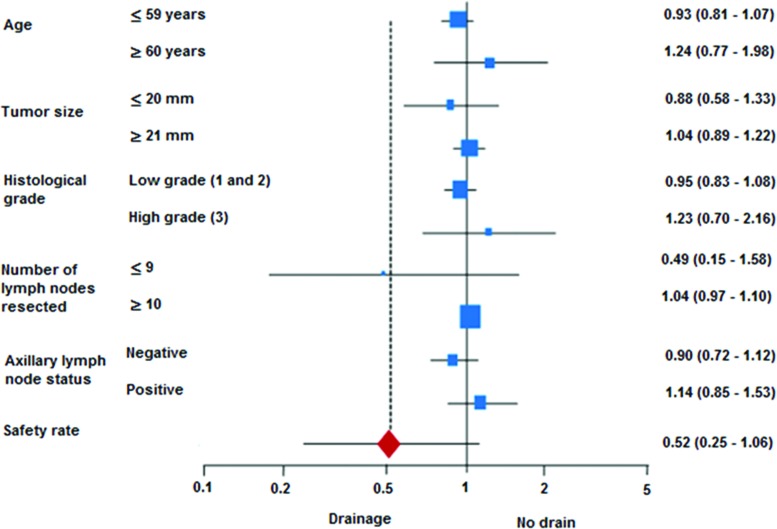
Forest plot of the effect of each individual group on the safety rate. The safety rate was defined as the total number of patients without complications divided by the total number of patients in each group.

The analysis of complications included the data from 179/240 patients, with the remaining 61 patients being lost to follow-up. Infections occurred in 25 cases (14%): 20 cases of hyperemia (11.2%) and 5 cases of abscess (2.8%). Wound dehiscence occurred in 15 cases (8.4%), necrosis in 4 cases (2.2%) and hematoma in 15 cases (8.4%). The two groups were significantly different in relation to wound dehiscence, which was more common in the no drain group. No other statistically significant differences were found between the two groups for any of the other complications registered (infection, necrosis or hematoma). The safety rate was similar for both groups, as shown in Table 3. Analysis of the individual subgroups showed that none of the control variables had any effect on safety (Figure 1).

## DISCUSSION

Investigation of sentinel lymph nodes has been the routine technical approach for dealing with the axillae of patients with early stage breast cancer 14,15. Nevertheless, axillary dissection with drainage continues to be performed during surgery in a large number of cases because of compromised lymph nodes or in cases in which identification of the sentinel lymph node proves impossible. Such procedures involve adverse events, with seroma formation constituting the principal complication, followed by infection, necrosis, dehiscence and hematoma 1,16.

Systematic use of suction drainage has been performed in an attempt to reduce the rates of seroma formation. Among breast cancer patients undergoing surgery, this rate can be as high as 90% 16,17. However, over recent years, the possibility of not draining the axilla has been studied 11,12 since seroma formation rates tend to remain very high even with the use of drains 18. This complication occurs secondary to the disruption of lymph channels, an inevitable consequence of extensive surgical dissection and disruption of tissue planes, creating a dead space 18.

To the best of our knowledge, the results of the other randomized studies comparing drainage versus no drainage have been inconsistent, and furthermore, the previous studies also differ in some points from the results of the present study. Classe et al. reported the results of breast-conserving surgeries 19, while Purushotham et al. reported on mastectomies and breast-conserving surgeries, with both groups performing axillary dissection only at levels I and II 20, whereas in the present study, axillary dissection was performed at all three levels. On the other hand, although axillary dissection was performed at all three levels in the study conducted by Soon et al., those investigators also included patients submitted to mastectomy 16.

In previously published randomized clinical trials on breast cancer surgery in which the use of drainage was compared with no drainage, and axillary dissection was performed both in cases of mastectomy and in breast-conserving surgery, no statistically significant differences were found between the groups insofar as seroma formation was concerned 16,20,21.

In the present study, the rate of seroma formation was 44%, and unlike previous studies 11, seroma formation was around four times greater in the no drain group, a difference that was statistically significant. The mean volume of serous fluid drained was much greater in the group of women in whom drainage was performed, and the mean volume of fluid aspirated was much greater in the no drain group. However, when the total volume of fluid was analyzed, i.e., the volume of fluid from drainage added to the volume of aspirated fluid, there was no statistically significant difference between the groups.

The mean number of needle aspirations was significantly greater in the no drain group and may be considered a sign of the severity of a seroma. The number of aspirations performed in the present study ranged from 0 to 7, a range that is similar to that reported from other series, such as the study conducted by Gonzalez et al., who performed between 1 and 6 aspirations after removal of the drain in patients submitted to breast cancer surgery with axillary dissection 22. These aspirations also generate additional costs and can reduce these patient’s postsurgical quality of life.

According to these results, drainage does not prevent seroma formation. The fluid that is not drained will have to be aspirated by needle aspiration. The present findings are in agreement with results published by other investigators, such as Soon et al., who reported total fluid volumes of 538.8 ml in the drained group *versus* 856.7 ml in the no drain group 16.

With regard to the other complications, the infection rate in the present study was 14%, 11.2% of which consisted of cellulitis that receded with the use of antibiotics. The remaining 2.8% consisted of abscesses. There was no statistically significant difference between the two groups. Hall and Hall 23 reported a rate of cellulitis of 11% and a rate of abscesses of 4.6% following breast surgery, a result that was similar to that found in the present study. Therefore, care should be taken to ensure that antisepsis of the surgical area is strictly observed.

Comparison of the two study groups showed infection rates of 12.2% and 15.5% for the drainage and no drain groups, respectively. The infection rates detected in previous randomized clinical trials differed greatly, with reports of 1.9% 21 and 25% 16. As in the present study, none of the other authors observed any significant differences in the rate of infection between the drainage and no drain groups of patients 3,16,21. Nevertheless, in 2005, Soon et al. reported a tendency for infection in the group of women subjected to drainage, although patients submitted to mastectomy were also included in that study 16.

The rate of wound dehiscence in the present study was 8.4%, which was higher than rates reported from other series 3. This complication was strongly influenced by the no drain group, in which the rate was 13.5% versus 2.4% in the drainage group. If we consider only the data from the drainage group, then the rate encountered was similar to rates reported from other previously published studies 1,6,23.

In the present study, the rate of necrosis in the skin flaps was 2.2%, which is similar to the rate of 2.35% reported by Chintemani et al. 24. On the other hand, the rate of hematoma of 8.4% found in the present study was higher than rates reported by other groups, which ranged from 2.1% to 3.9% 1,3; however, the presence or absence of a drain did not affect these rates.

In this study, the total number of patients with no complications (the safety rate) was similar in both groups; however, there was a tendency toward greater numbers of complications in the no drain group, influenced by the greater number of cases of dehiscence. These data are in agreement with the findings by Tylor et al., who also failed to find any difference between the two groups insofar as the overall number of complications was concerned 11. In the exploratory analysis, the individual subgroups exerted no effect on the safety rate.

The results presented here allow the possible alternatives to be discussed with patients, i.e., the inconvenience of drainage versus a greater number of needle aspirations, with the greater potential risk of wound dehiscence. Patients are thus able to participate with the physicians in the decision-making process regarding their treatment.

The safety rate (i.e., the total number of patients with no complications) was similar in both study groups, irrespective of whether or not axillary drainage was used. Therefore, axillary lymph node dissection can be performed safely without axillary drainage; however, there is a greater possibility of wound dehiscence, and a greater number of needle aspirations are required for patients who do not undergo auxiliary drainage.

## AUTHOR CONTRIBUTIONS

Ribeiro LF and Freitas-Junior R wrote and revised the manuscript. Freitas-Junior R, Ribeiro LF, Moreira MA, Queiroz GS, Esperidião MD, Silva MA, Pereira RJ, Zampronha RA, Rahal RM, Soares LR, Santos DL, Thomazini MV, Faria CF and Paulinelli RR were responsible for the clinical trial and for revising the manuscript.

## Figures and Tables

**Table 1:** Demographic analysis comparing the group in which axillary drainage was performed with the group in which this procedure was not performed.*

	Drainage n=119	No drainage n=121	*p*-value
Mean age (± standard deviation)	49.38 (± 10.95)	49.91 (±12.86)	0.87
Median tumor size (25^th^ – 75^th^ percentile)	30.00 (20.00 – 40. 00)	28.00 (20.00 – 40.00)	0.89
Median histological grade (25^th^ – 75^th^ percentile)	2.00 (2.00 – 3.00)	2.00 (2.00 – 3.00)	0.65
Median number of lymph nodes resected (25^th^ – 75^th^ percentile)	17.00 (13.00 – 21.00)	17.00 (14.00 – 21.00)	0.46
Median number of positive lymph nodes (25^th^ – 75^th^ percentile)	0.00 (0.00 – 2.00)	0.00 (0.00 – 2.00)	0.49

* Mann-Whitney test/Student’s t-test.

**Table 2:** Comparison of the volume of drained and aspirated serous fluid in the drainage and no drain groups.*

	Drainage		No drain		
	Median (25^th^ – 75^th^ percentile)	Mean (± SD)	Median (25^th^ – 75^th^ percentile)	Mean (± SD)	*p*-value
Volume drained (ml)	500.00 (233.50 – 864.00)	581.80 (442.60)	0.00 (0.00 – 0.00)	21.50 (107.90)	<0.01
Volume aspirated (ml)	0.00 (0.00 – 270.00)	176.60 (298.20)	522.50 (130.00 – 1148.80)	858.40 (1434.50)	<0.01
Number of aspirations	0.50 (0.00 – 4.00)	2.10 (3.10)	5.00 (3.00 – 7.00)	5.90 (6.20)	<0.01
Total drained (ml)	720.00 (395.00 – 1145.00)	779.30 (451.90)	540.00 (215.00 – 1152.50)	890.60 (1434.30)	0.19

* Mann-Whitney test/Student’s t-test.

**Table 3:** Analysis of the complications observed according to group (axillary drainage or no drain).

	Drainage n (%)	No drain n (%)	RR (95%CI)	*p*-value
Infection	10 (12.20%)	15 (15.50%)	1.17 (0.70-1.94)	0.53
Dehiscence	2 (2.40%)	13 (13.50%)	3.70 (1.01-13.59)	<0.01
Necrosis	1 (1.20%)	3 (3.10%)	1.87 (0.34-10.31)	0.39
Hematoma	6 (7.20%)	9 (9.40%)	1.17 (0.62-2.23)	0.61
Safety rate	67 (81.70%)	68 (70.10%)	0.52 (0.25-1.06)	0.07

RR = relative risk;

CI = confidence interval. Safety rate: total number of patients without complications divided by the number of patients in each group.

## References

[1] Freitas-Júnior R, Oliveira EL, Pereira RJ, Silva MA, Esperidião MD, Zampronha RA (2006). Modified radical mastectomy sparing one or both pectoral muscles in the treatment of breast cancer: intra and postoperative complications. Sao Paulo Med J.

[2] Jagsi R, Jiang J, Momoh AO, Alderman A, Giordano SH, Buchholz TA (2016). Complications after mastectomy and immediate breast reconstruction for breast cancer: A claims-based analysis. Ann Surg.

[3] Thomson DR, Sadideen H, Furniss D (2013). Wound drainage after axillary dissection for carcinoma of the breast. Cochrane Database Syst Rev.

[4] Mastrella Ade S, Freitas-Junior R, Paulinelli RR, Soares LR (2014). Incidence and risk factors for winged scapula after surgical treatment for breast cancer. J Clin Nurs.

[5] Nesvold IL, Reinertsen KV, Fosså SD, Dahl AA (2011). The relation between arm/shoulder problems and quality of life in breast cancer survivors: a cross-sectional and longitudinal study. J Cancer Surviv.

[6] Kottayasamy Seenivasagam R, Gupta V, Singh G (2013). Prevention of seroma formation after axillary dissection – a comparative randomized clinical trial of three methods. Breast J.

[7] Catsman CJ, Beek MA, Rijken AM (2016). Talc seromadesis in patients with chronic seroma formation after breast surgery. Springerplus.

[8] Sajid MS, Hutson KH, Rapisarda IF, Bonomi R (2013). Fibrin glue instillation under skin flaps to prevent seroma-related morbidity following breast and axillary surgery. Cochrane Database Syst Rev.

[9] Srivastava V, Basu S, Shukla VK (2012). Seroma formation after breast cancer surgery: what we have learned in the last two decades. J Breast Cancer.

[10] van Bastelaar J, Beckers A, Snoeijs M, Beets G, Vissers Y (2016). Flap fixation reduces seroma in patients undergoing mastectomy: a significant implication for clinical practice. World J Surg Oncol.

[11] Taylor JC, Rai S, Hoar F, Brown H, Vishwanath L (2013). Breast cancer surgery without suction drainage: the impact of adopting a ‘no drains' policy on symptomatic seroma formation rates. Eur J Surg Oncol.

[12] Ebner F, deGregorio N, Vorwerk E, Janni W, Wöckel A, Varga D (2014). Should a drain be placed in early breast cancer surgery. Breast Care.

[13] Freitas-Junior R, Cavalcante AF, Soares LR, Pádua AP, Sousa PT, Ribeiro LF (2014). Estudo comportamental sobre a drenagem axilar no câncer de mama. Rev Bras Mastologia.

[14] Giuliano AE, Ballman K, McCall L, Beitsch P, Whitworth PW, Blumencranz P (2016). Locoregional recurrence after sentinel lymph node dissection with or without axillary dissection in patients with sentinel lymph node metastases: long-term follow-up from the American College of Surgeons Oncology Group (Alliance) ACOSOG Z0011 Randomized Trial. Ann Surg.

[15] Truin W, Roumen RM, Siesling S, van der Heiden-van der Loo M, Lobbezoo DJ, Tjan-Heijnen VC (2016). Sentinel lymph node biopsy and isolated tumor cells in invasive lobular versus ductal breast cancer. Clin Breast Cancer.

[16] Soon PS, Clark J, Magarey CJ (2005). Seroma formation after axillary lymphadenectomy with and without the use of drains. Breast.

[17] Nadkarni MS, Rangole AK, Sharma RK, Hawaldar RV, Parmar VV, Badwe RA (2007). Influence of surgical technique on axillary seroma formation: a randomized study. ANZ J Surg.

[18] Ouldamer L, Caille A, Giraudeau B, Body G (2015). Quilting suture of mastectomy dead space compared with conventional closure with drain. Ann Surg Oncol.

[19] Classe JM, Berchery D, Campion L, Pioud R, Dravet F, Robard S (2006). Randomized clinical trial comparing axillary padding with closed suction drainage for the axillary wound after lymphadenectomy for breast cancer. Br J Surg.

[20] Purushotham AD, McLatchie E, Young D, George WD, Stallard S, Doughty J (2002). Randomized clinical trial of no wound drains and early discharge in the treatment of women with breast cancer. Br J Surg.

[21] Ruggiero R, Procaccini E, Piazza P, Docimo G, Iovino F, Antoniol G (2008). Effectiveness of fibrin glue in conjuntion with collagen patches to reduce seroma formation after axillary lynphadenectomy for breast cancer. Am J Surg.

[22] Gonzales EA, Saltzstein EC, Riedner CS, Nelson BK (2003). Seroma formation following breast cancer surgery. Breast J.

[23] Hall JC, Hall JL (2004). The measurement of wound infection after breast surgery. Breast J.

[24] Chintamani, Singhal V, Singh J, Bansal A, Saxena S (2005). Half versus full vacuum suction drainage after modified radical mastectomy for breast cancer – a prospective randomized clinical trial [ISRCTN24484328]. BMC Cancer.

